# Therapeutic efficacy of AAV8-mediated intrastriatal delivery of human cerebral dopamine neurotrophic factor in 6-OHDA-induced parkinsonian rat models with different disease progression

**DOI:** 10.1371/journal.pone.0179476

**Published:** 2017-06-16

**Authors:** Lizheng Wang, Zixuan Wang, Rui Zhu, Jinpeng Bi, Xinyao Feng, Wenmo Liu, Jiaxin Wu, Haihong Zhang, Hui Wu, Wei Kong, Bin Yu, Xianghui Yu

**Affiliations:** 1 National Engineering Laboratory for AIDS Vaccine, School of Life Sciences, Jilin University, Changchun, Jilin Province, China; 2 Key Laboratory for Molecular Enzymology and Engineering, the Ministry of Education, School of Life Sciences, Jilin University, Changchun, Jilin Province, China; University of Nebraska Medical Center, UNITED STATES

## Abstract

Parkinson’s disease (PD) is a progressive and age-associated neurodegenerative disorder. Patients at different stages of the disease course have distinguished features, mainly in the number of dopaminergic neurons. Cerebral dopamine neurotrophic factor (CDNF) is a recently discovered neurotrophic factor, being deemed as a hopeful candidate for PD treatment. Here, we evaluated the efficacy of CDNF in protecting dopaminergic function using the 6-OHDA-induced PD rat model suffering from different levels of neuronal loss and the recombinant adeno-associated virus 8 (AAV8) as a carrier for the *CDNF* gene. The results showed that AAV8-CDNF administration significantly improved the motor function and increased the tyrosine hydroxylase (TH) levels in PD rats with mild lesions (2 weeks post lesion), but it had limited therapeutic effects in rats with severe lesions (5 weeks post lesion). To better improve the recovery of motor function in severely lesioned PD rats, we employed a strategy using the *CDNF* gene along with the *aromatic amino acid decarboxylase* (*AADC*) gene. This combination therapeutic strategy indeed showed an enhanced benefit in restoring the motor function of severely lesioned PD rats by providing the neuroprotective effect of CDNF and dopamine enhancing effect of AADC as expected. This study may provide a basis for future clinical application of CDNF in PD patients at different stages and offer a new alternative strategy of joint use of CDNF and AADC for advanced PD patients in clinical trials.

## Introduction

Parkinson’s disease (PD), the second most common neurodegenerative disease after Alzheimer's, affects elderly people worldwide [1]. A primary pathological hallmark of PD is the loss of nigrostriatal dopamine neurons, directly resulting in the motor symptoms of PD patients, including resting tremor, rigidity, akinesia and postural instability. Current clinical therapeutic strategies with oral medications and continuous electrical stimulation of basal ganglia structures (DBS) cannot mediate long-term effects. Moreover, oral medications invariably cause side-effects, such as levodopa-induced dyskinesias (LIDs) [2, 3]. Therefore, a gene therapy-based strategy may provide a superior innovative method for treating PD.

Dopamine enhancing gene therapy and neurotrophic factor gene therapy are the main strategies in current pre-clinical and clinical studies of gene therapy for PD [4]. Dopamine enhancing gene therapy typically increases the dopamine levels in the brain by overexpressing the related enzymes during dopamine synthesis, such as tyrosine hydroxylase (TH), GTP cyclohydrolase 1 (GCH1) and aromatic amino acid decarboxylase (AADC). Both adeno-associated virus serotype 2 (AAV2)-mediated human AADC expression [5, 6] and lentivirus (LV)-mediated TH-AADC-GCH1 co-expression have been shown to be effective in alleviating symptoms of PD patients in clinical trials [7]. However, delivery of dopamine synthesis-related enzyme genes failed to prevent the neurodegeneration to alter the PD progression. Thus, neurotrophic factor gene therapy may be the most hopeful strategy to prevent disease progression by protecting and even restoring dopaminergic neurons [8].

Neurotrophic factors, naturally occurring proteins that are critical to neuronal differentiation and maturation during development and adulthood, are promising candidates for the neuroprotective or even neurorestorative treatment of PD [8]. Among them, glial-derived neurotrophic factor (GDNF) [9, 10] and its structural homolog neurturin (NRTN) [11, 12] are well-established to be effective in protecting dopaminergic neurons from several insults and restoring function in animal models of PD. However, both GDNF [13–16] and NRTN [16–18] have shown only modest effects in clinical trials. Encouragingly, the discovery of cerebral dopamine neurotrophic factor (CDNF) provides a new alternative. CDNF is a member of the mesencephalic astrocyte-derived neurotrophic factor (MANF) protein family [19, 20], the structure of which is different from GDNF. *CDNF* mRNA can be detected in various brain tissues including the striatum in both embryonic and adult mice [21]. Infusion of Sf9–derived recombinant human CDNF proteins into the rat brain has been shown to prevent 6-OHDA-induced dopaminergic neural degeneration in a rat model of PD or MPTP-induced PD animal models [21–24].

Initial studies [21–23] demonstrated the ability of CDNF to protect PD rats with mild lesions from neurodegeneration, but more studies are needed to explore the efficacy of CDNF in late PD. In this study, we constructed a recombinant adeno-associated virus 8 carrying CDNF gene (AAV8-CDNF) and sought to directly compare the therapeutic effects of AAV8-CDNF in 6-OHDA induced PD rat models with different levels of neuronal loss. Due to the insufficient protection observed in PD rats with severe lesions in the experiments, we then tested whether a joint delivery of CDNF and AADC, an enzyme which can convert levodopa to dopamine, could ameliorate the motor dysfunction in this advanced PD model. A combination of neuroprotective effect provided by CDNF and AADC that accelerated dopamine synthesis was expected to better improve the behavior of severely lesioned PD rats. The results may offer a foundation for future clinical applications of CDNF.

## Materials and methods

### Therapeutic experimental design

To test the therapeutic effect of rAAV8 vector-mediated delivery of CDNF expression in PD rats at different stages of disease progression, we carried out animal experiments as follows. First, 6-OHDA was injected into the right side of the brain of rats, which were then divided into 3 groups (n = 14–16 per group), excluding those rotated less than 40 turns per hour after apomorphine administration (2mg/kg) at day 10 after the injection. One group was administered AAV8-CDNF at week 2 post lesion as the early-treated group. Another group was administered AAV8-CDNF at week 5 post lesion as the late-treated group. Rats in yet another group were administered an AAV8-RFP viral vector at week 2 post lesion as a control. The injection sites of 6-OHDA and viral vectors are shown in S1 Fig. Drug-induced rotational behavior was detected at two-week intervals, and immunochemistry was employed for TH analysis at week 17 post lesion. The schematic representation of experimental schedules is shown in S2 Fig.

### Animals

Adult male Wistar rats were used for all experiments. They were housed three per cage under a 12 h light/dark cycle in a temperature-controlled room (21–23°C). Food and water were available *ad libitum*. All animals used in the procedures were handled in strict accordance with the National Institutes of Health Guide for the Care and Use of Laboratory Animals, and the procedures were approved by the University Committee on the Use and Care of Animals of Jilin University of China. All surgeries were performed under anesthesia, and all efforts were made to minimize suffering of animals.

### Production of rAAV vectors

The cDNA encoding the human *CDNF* gene, *AADC* gene or *red fluorescent protein* (*RFP*) gene was cloned into the pAAV-CAG-MCS-WPRE vector. The processes of AAV packaging and purification were previously reported [25]. The titers of AAV8-CDNF, AAV8-AADC and AAV8-RFP viral vectors were 2.8 × 10^13^ viral genomes/mL (vg/mL), 2.2 × 10^13^ vg/mL and 1.8 × 10^13^ vg/mL, respectively. All primary plasmids for rAAV8 packaging were obtained from AXYBIO (Changsha, China).

### 6-OHDA-induced PD rat model

Rats weighing 240–300 g were anesthetized with intraperitoneally (i.p.) administered pentobarbital sodium (60 mg/kg). The 6-OHDA (4 μg 6-OHDA dissolved in 4 μL sterile saline containing 0.02% ascorbic acid) was unilaterally injected into the right hemisphere (coordinates from bregma: AP, -4 mm; ML, ^+^1.65 mm; DV, -8 mm) according to the Paxinos and Watson's rat brain atlas (1998) with a Hamilton syringe (0.46 mm in diameter, blunt tip) at a rate of 0.5 μL per minute. The needle was kept in place for 8 min and then slowly withdrawn over a 3 to 4 min period.

### Administration of rAAV8 vectors

AAV8-RFP, AAV8-CDNF, AAV8-AADC or the mixture of AAV8-CDNF/AAV8-AADC was unilaterally injected into the right striatum using two coordinates relative to the bregma and dura: (A/P + 0.5 mm, L/M +2.0 mm, D/V -5.0 mm) and (A/P + 0.5 mm, L/M +3.2 mm, D/V -5.0 mm). In our previous comparison, the transgene was expressed and spread more efficiently using the two neighboring injection sites in the striatum than using a single injection site (data not shown). The titer of each of the rAAV8 vectors microinjected into the rat brain was 2 × 10^10^ vg (in 2 μL PBS) throughout this study. The syringe was left in place for 8 min and then raised slowly out of the brain over 2 min. The early administration of rAAV8 was performed at 2 weeks post injection of 6-OHDA, and the late administration of rAAV8 was conducted at 5 weeks post lesion.

### Detection of RFP expression in the rat brain

Animals were microinjected with AAV8-RFP viral vectors using the two neighboring sites mentioned above. At different time points post injection, three rats were killed, and each brain was isolated and placed in cold PBS immediately. Within 15 min, RFP expression was observed by using an In Vivo Imaging System Fx Pro (Kodak, USA).

### Behavioral tests

The tendency of rats to rotate in response to apomorphine (Sigma) was tested in automatic rotometer bowls (Med Associates, Inc., GA, USA). Following a habituation period of 30 min, a single dose of apomorphine (2 mg/kg, i.p.) was administrated to check rotational response. Apomorphine causes rats to characteristically turn to the contralateral side when the supersensitive receptors in the lesioned side of the brain are activated. The number of contralateral rotations was counted for 60 min.

### Tissue collection and dopamine detection

At the end of the experiment, the late-treated animals were killed by decapitation while still anesthetized with pentobarbital sodium. The brains were removed rapidly and chilled in ice-cold saline. The brains were separated into left and right hemispheres, and the striatum was dissected from each half of the brain as a single piece. The tissue pieces were placed in pre-weighed vials, weighed and frozen in liquid nitrogen. Samples were stored at -80°C until assayed by high performance liquid chromatography (HPLC).

For determining dopamine content, the samples were sonicated in 300 μL of cold 0.1 M perchloric acid containing dihydroxybenzylamine as an internal standard. After centrifuging samples for 5 min at 13,500 × *g*, the supernatant was diluted with HPLC mobile phase and injected (50 ml) onto the HPLC column. The mobile phase consisted of sodium acetate (0.1 M), EDTA (0.1 mM) and methanol (10%) and was adjusted to pH 5.1 with glacial acetic acid, filtered and degassed. A C18 column (150 mm × 4.6 mm i.d., 5 μm) was used for separations with a flow rate of 0.7 ml/min.

### Immunohistochemistry

Rats were anesthetized and perfused with saline and 4% paraformaldehyde in 0.01 M PBS. Brains were removed, post-fixed in 4% paraformaldehyde overnight and then dehydrated with 20–30% gradient sucrose–PBS solutions. Brain tissues were frozen in isopentane and kept at −80°C for further immunohistochemistry procedures.

Coronal brain sections (30-μm thickness) from the entire substantia nigra pars compacta (SNpc) and a large part of the striatum were prepared on a slicing vibratome and collected in a 6-well plate. Every 6th section of a brain was collected in a well. Sections containing the SNpc and striatum were selected according to Paxinos & Watson's rat brain atlas, with the rostro-caudal extent of the SNpc being between −4.8 mm and −6.12 mm and the rostro-caudal extent of the striatum being between ^+^2.16 mm and −0.24 mm with respect to the bregma. Free-floating staining procedures were conducted to detect TH and CDNF using immunoperoxidase. Briefly, sections were placed in citrate buffer to retrieve antigens, washed in PBS-Triton X-100 (0.2%) for 3 × 10 min and then rinsed in 0.3% hydrogen peroxide to quench the endogenous peroxide. Appropriate primary antibodies [mouse anti-TH (diluted 1:200, Sigma) or rabbit anti-CDNF (diluted 1:500, Abcam) or anti-AADC (diluted 1:1000, Merk Millipore)] were incubated with sections at 4°C overnight. The horseradish peroxidase (HRP)-labeled secondary antibodies [goat anti-mouse IgG/HRP (diluted 1:500, ZSGB-BIO) or goat anti-rabbit IgG/HRP (diluted 1:500, ZSGB-BIO)] were developed by a peroxidase reaction with diaminobenzidine (DAB) as the chromagen.

### Quantification of TH-reactive fibers and TH^+^ neurons

The density of TH-reactive fibers was measured using Image pro-plus software (Media Cybernetics, Bethesda, MD, USA) along a line drawn across the dorsal part of the striatum. All density values were corrected for the background density. At least 4 sections from the striatum of each rat brain were analyzed. The results are given as a percentage of the lesioned striatum to the intact striatum.

To quantify the number of TH^+^ neurons in SNpc, an optical dissector method of stereological analysis was conducted using Stereo Investigator Software (MicroBrightField, Williston, VT, USA). A fractionator probe was established for each section, and six sections in total covering the entire SN were examined. The results are given as the percentage of TH^+^ neurons compared to the intact side.

### Statistical analysis

Upaired t-tset or one-way analysis of variance (ANOVA) followed by the Newman–Keuls test or two-way ANOVA was performed to analyze the data. The results are expressed as the mean ± S.E.M. and are considered significant at *P* < 0.05.

## Results

### rAAV8-mediated transgene expression in the rat striatum

**Fig 1:**
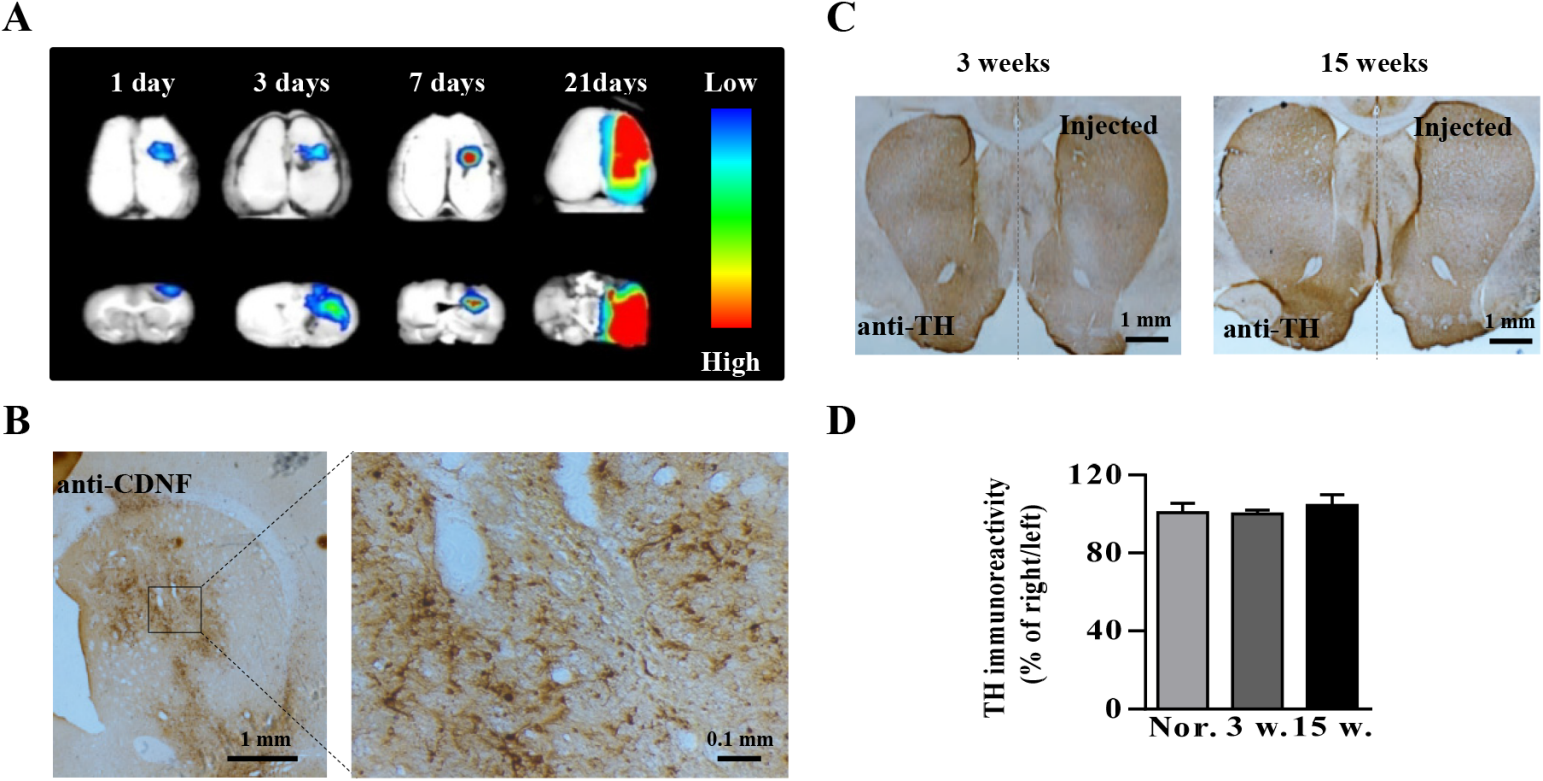
rAAV8-mediated transgene expression and influence of AAV8-CDNF in striatal TH expression of rats. (A) RFP expression was detected at 1 day, 3 days, 7 days and 21 days post AAV8-RFP injection. Three rats were analyzed at each time-point, and the representative images are shown. Images in the upper row were taken from the complete brain. The lower row shows images of brains sliced in coronary. All of the slices included the striatum. (B) AAV8-CDNF-mediated CDNF expression in the striatum was detected by immuno-histochemical staining at 2 weeks post viral injection. (C) TH expression was detected by immuno-histochemical staining at week 3 and week 15 post AAV8-CDNF injection. Three rats were analyzed at per time-point, and representative images are shown. The labels ‘Nor.’, ‘3 w.’ and ‘15 w.’ represent normal rats, rats at week 3 post AAV8-CDNF administration and rats at week 15 post AAV8-CDNF administration, respectively. No significant difference was found between the three groups.

To ensure that the rAAV8 vectors were infectious and able to mediate efficient foreign gene expression, the AAV8-RFP vectors carrying *RFP* as a reporter gene were injected into the rat striatum for detection of transgene expression. The AAV8-RFP-mediated RFP expression was observed on day 1, 3, 7 and 21 post viral injection (Fig 1A). The expression of RFP was observed after 24 h post viral delivery, and it increased over time to a high level and was widely distributed in the injected hemisphere over a 21-day period. These results indicated that the rAAV8-mediated transgene expression in the brain was rapid, efficient and widely spread to achieve a good gene therapy effect. Thus, we chose the rAAV8 vector to deliver the *CDNF* gene to treat PD rats in the subsequent experiment. CDNF was well-expressed in the rat brain after two weeks post AAV8-CDNF injection (Fig 1B).

At week 3 and week 15 post AAV8-CDNF delivery into the striatum of normal rats, the TH expression was detected in order to evaluate the influence of overexpression of this protein. The results showed that CDNF overexpression mediated by the rAAV8 vector did not downregulate TH expression (Fig 1C). AAV8-AADC vectors used in the combination therapeutic strategy for treating severely lesioned rats also mediated efficient AADC expression in the striatum (S2 Fig).

### TH expression and drug-induced rotational behavior at different time points post lesion by 6-OHDA administration

**Fig 2:**
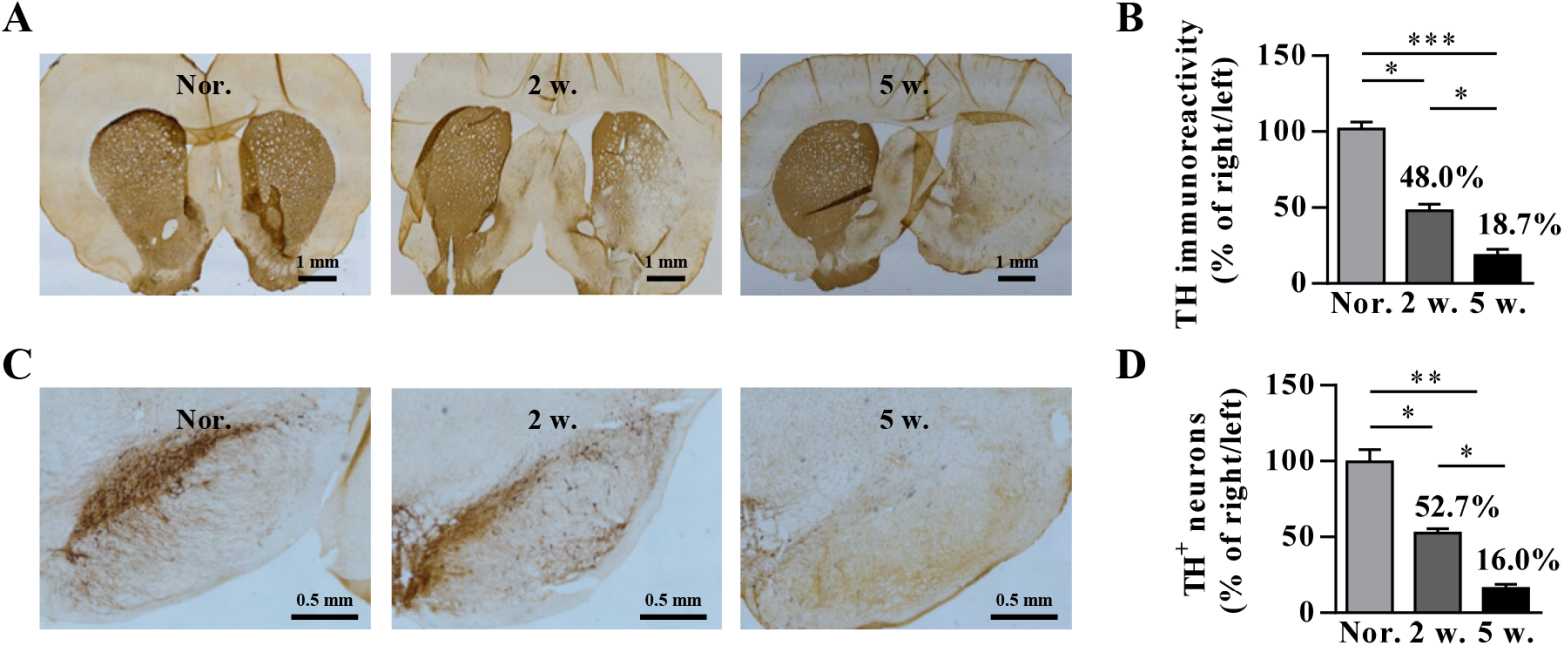
Immunostaining for TH in the striatum and SN at different time-points post 6-OHDA lesion. Three rats were employed per time-point, and representative images are shown. (A) Representative TH-immunostaining images in striatum are shown. (B) Quantification of TH immunoreactivity in the striatum. (C) Representative images of TH immunostaining of the SNpc. (D) Quantification of TH^+^ neurons in the SNpc. ‘Nor.’, ‘2 w.’ and ‘5 w.’ represent normal rats, rats at week 2 post lesion and rats at week 5 post lesion, respectively. Significance differences were calculated by one-way ANOVA in (B) and (D). *P < 0.05, **P < 0.005, ***P < 0.001.

The expression of TH by immunochemistry in the striatum and SNpc was detectedafter 6-OHDA lesion. We found that the TH expression in the brain of the 6-OHDA-induced PD rat model was reduced to different levels at 2 weeks and 5 weeks post lesion (Fig 2). The proportion of TH^+^ fibers in the striatum was approximately 48.0% and that of TH^+^ neurons was 52.7% at 2 weeks after the 6-OHDA injection, compared with the non-lesioned side of the brain. At five weeks post lesion, the level of TH expression in the striatum and SNpc was less than 20.0%. Therefore, we used PD rats at 2 weeks or 5 weeks post lesion for the subsequent experiments.

The rotational behavior was tested every week post 6-OHDA lesion. At the first 2 tests, rats showed slight and non-standard rotational behavior after high-dose apomorphine administration, but the rotations of 6-OHDA lesioned rats could be recorded using automatic rotometer bowls; at week 3 post lesion, rats began to rotate more, and the rotations became relatively stable at week 4, 5 and 6 (S3 Fig).

### Rotational behavior of PD rats after AAV8-CDNF administration

**Fig 3:**
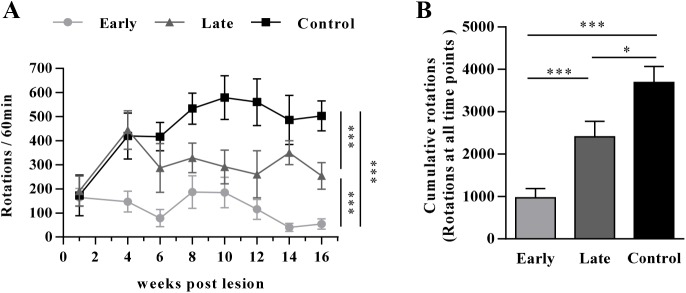
Rotation behavior test of early-treated rats and late-treated rats. (A) Rotation cycles of rats at detection points (n = 14–16). ‘Early’ represents the group receiving early treatment with AAV8-CDNF at week 2 post lesion. ‘Late’ represents the late treatment group. ‘Control’ represents the group with AAV8-RFP administration. Two-way ANOVA was performed for analyzing the significant differences between groups. ***P < 0.001. (B) Cumulative rotations of rats in three groups at all detection points are shown. *P < 0.05, ***P < 0.001. One-way ANOVA was used to analyze the data. *P < 0.05, ***P < 0.001.

The drug-induced rotational behaviors of early-treated and late-treated PD rats are shown in Fig 3A. The administration of AAV8-CDNF was found to restore the motor function of both mildly lesioned PD rats and severely lesioned PD rats compared with the AAV8-RFP group, although to different extents. Drug-induced rotation in the rats was reduced at the first behavioral test. The 6-OHDA-induced rotational behavior of rats was alleviated to a significantly greater extent in the early-treated group compared with the late-treated group (*P* < 0.001). In the last test, rats in the early-treated group rotated 54 turns/60 min on average, while those in the late-treated group rotated more than 250 turns/60 min. The rotations of all rats in the early-treatment group were less than 100 turns; the rotations of 12 rats in the late-treatment group (14 rats) were mainly between 200 and 320 turns/60min; the rotations of 10 rats in the control group (14 rats) were more than 500 turns/60min and that of the others were more than 380 turns/60min. The cumulative rotational turns of all tested time points (Fig 3B) reflected the motor behavior during the whole experiment.

### Immunochemical analysis of TH in the rat brain after AAV8-CDNF administration

**Fig 4:**
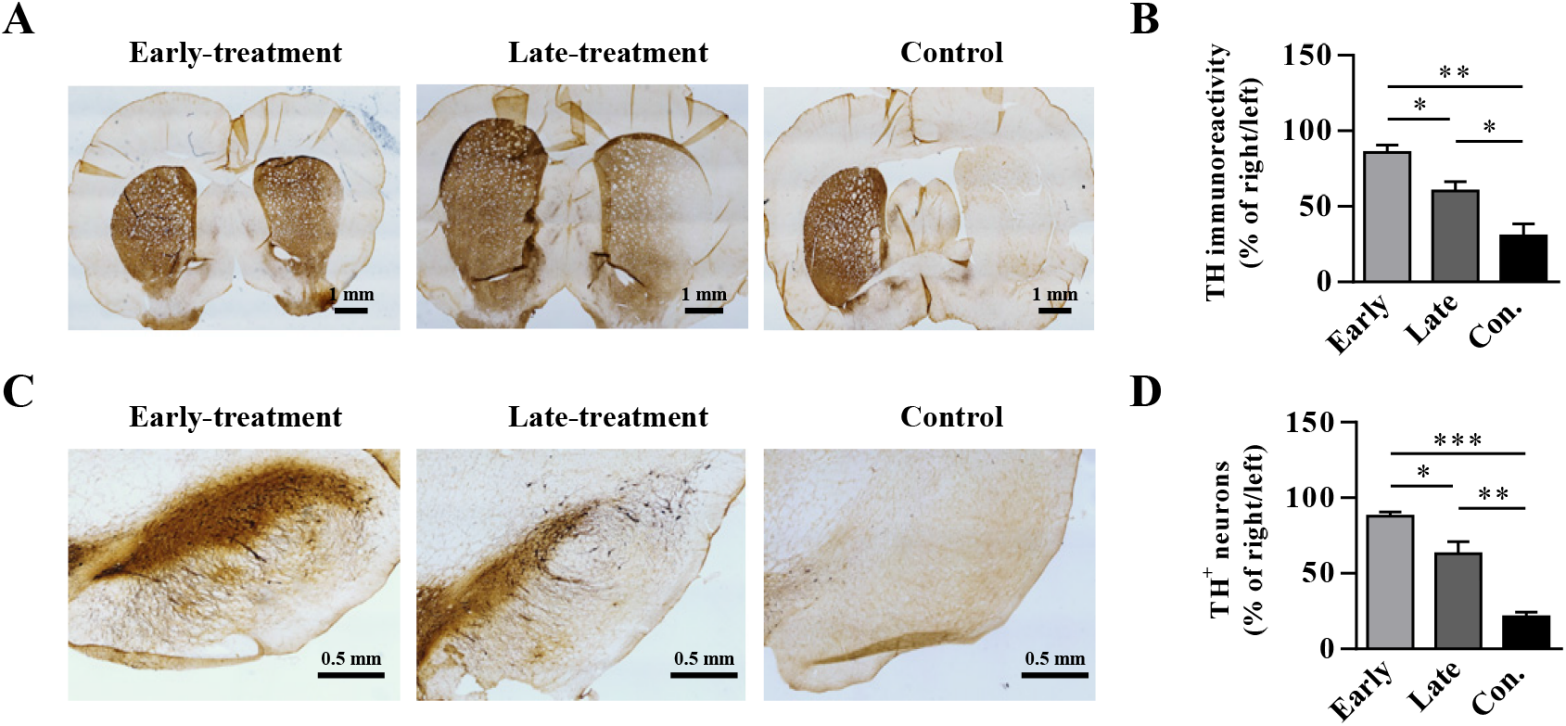
Immunostaining for TH in the striatum and SNpc of early-treated and late-treated rats. (A) Representative TH-immunostaining images in striatum from 5 rats/group detected are shown. (B) Quantification of TH immunoreactivity in the striatum. (C) Representative immunostaining images in SNpc are shown. (D) Quantification of TH^+^ neurons in the SNpc. ‘Early’ represents the group receiving early treatment with AAV8-CDNF at week 2 post lesion. ‘Late’ represents the late treatment group. ‘Con.’ represents the control group with AAV8-RFP administration. Significant differences in (B) and (D) were calculated by one-way ANOVA. *P < 0.05, **P < 0.005, ***P < 0.001.

CDNF can protect the dopaminergic system against 6-OHDA-induced neurotoxicity to prevent PD progression [21–23, 26–28]. We performed immunohistochemical staining of TH in the rat striatum and SNpc to evaluate the abilities of early and late administration of AAV8-CDNF to inhibit the progression of PD. Five rats reflecting the dominant level of rotations in each group were selected for TH analysis. Rotations of the selected rats are: early-treatment group, <100 turns /60min; late-treatment group, 200–320 turn/60min; control group, >500 turns/60min. The following groups are ordered according to their TH levels from greatest to least in the lesioned side both in the striatum (Fig 4A) and SNpc (Fig 4C): early-treated group, late-treated group and control AAV8-RFP group. Significant differences could be found between the early-treated group and late-treated group both in TH^+^ staining density of the striatum (Fig 4B, *P* < 0.05) and TH^+^ neuronal numbers in the SNpc (Fig 4D, *P* < 0.05). These results indicated that *CDNF* gene transfer by rAAV8 could better restore 6-OHDA-induced TH loss in the striatum and the SNpc and better prevent the disease progression when administered to PD rats with mild lesions, compared to when administrated to PD rats with severe lesions.

### Behavioral test of severely lesioned PD rats after combination therapy

**Fig 5:**
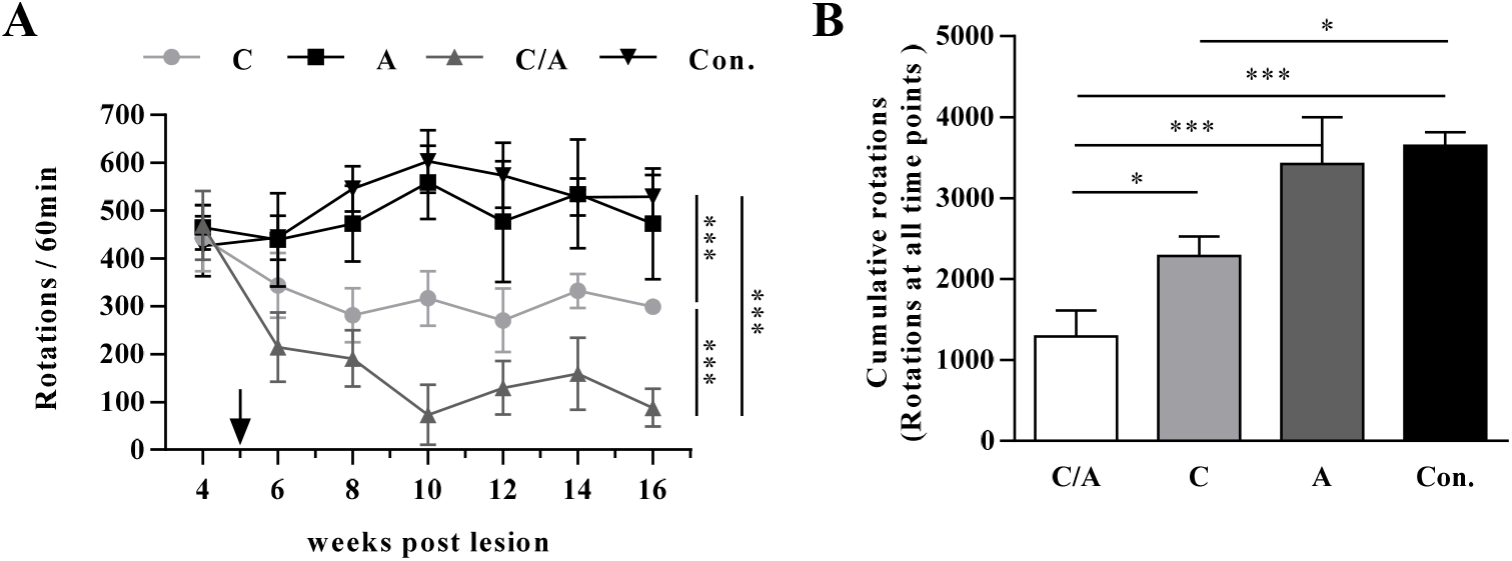
Rotational behavior test of PD rats at late stage of disease course with single-rAAV8 or combinational therapy. (A) Rats with rotational behavior were selected at week 4 post lesion and divided into 4 groups (n = 8–10). Two groups were administered AAV8-CDNF or AAV8-AADC alone, while another group was administered both viral vectors. The control group was administered AAV8-RFP. The arrow points to the time of treatment (5 weeks post lesion). Behavioral tests were carried out every two weeks. Two-way ANOVA was performed for analyzing the significant differences between groups. ***P < 0.001. (B) Cumulative rotations of rats in three groups at all detection points are shown. One-way ANOVA was used to analyze the data. *P < 0.05, ***P < 0.001. ‘C/A’, ‘C’, ‘A’ and, ‘Con.’ represent ‘CDNF/AADC group’, ‘CDNF group’, ‘AADC group’ and ‘control group with AAV8-RFP administration’, respectively.

Based on the limited therapeutic effect of AAV8-CDNF delivery in severely lesioned PD rats, we wanted to search for a new therapeutic strategy for motor improvement. A combination approach of using both AAV8-CDNF and AAV8-AADC was designed. The rats were divided into four groups at 4 weeks post lesion and administered rAAV8 vectors at the fifth week post lesion. Each group contained 8–10 rats. The drug-induced rotational behavior was tested at 2-week intervals as shown in Fig 5A, and the cumulative rotations of each group are shown in Fig 5B. The results showed that co-administration of AAV8-CDNF and AAV8-AADC or AAV8-CDNF alone could improve the rotation behavior of severely lesioned PD rats, compared with the control (AAV8-RFP) group, and the combination of AAV8-CDNF and AAV8-AADC resulted in the greatest extent of motor function recovery (*P* < 0.05, combination group *v*.*s*. AAV8-CDNF group). AAV8-AADC alone did not significantly reduce the rotation behavior of the severely lesioned PD rats.

### Analysis of TH and dopamine levels in severely lesioned PD rats after combination therapy

**Fig 6:**
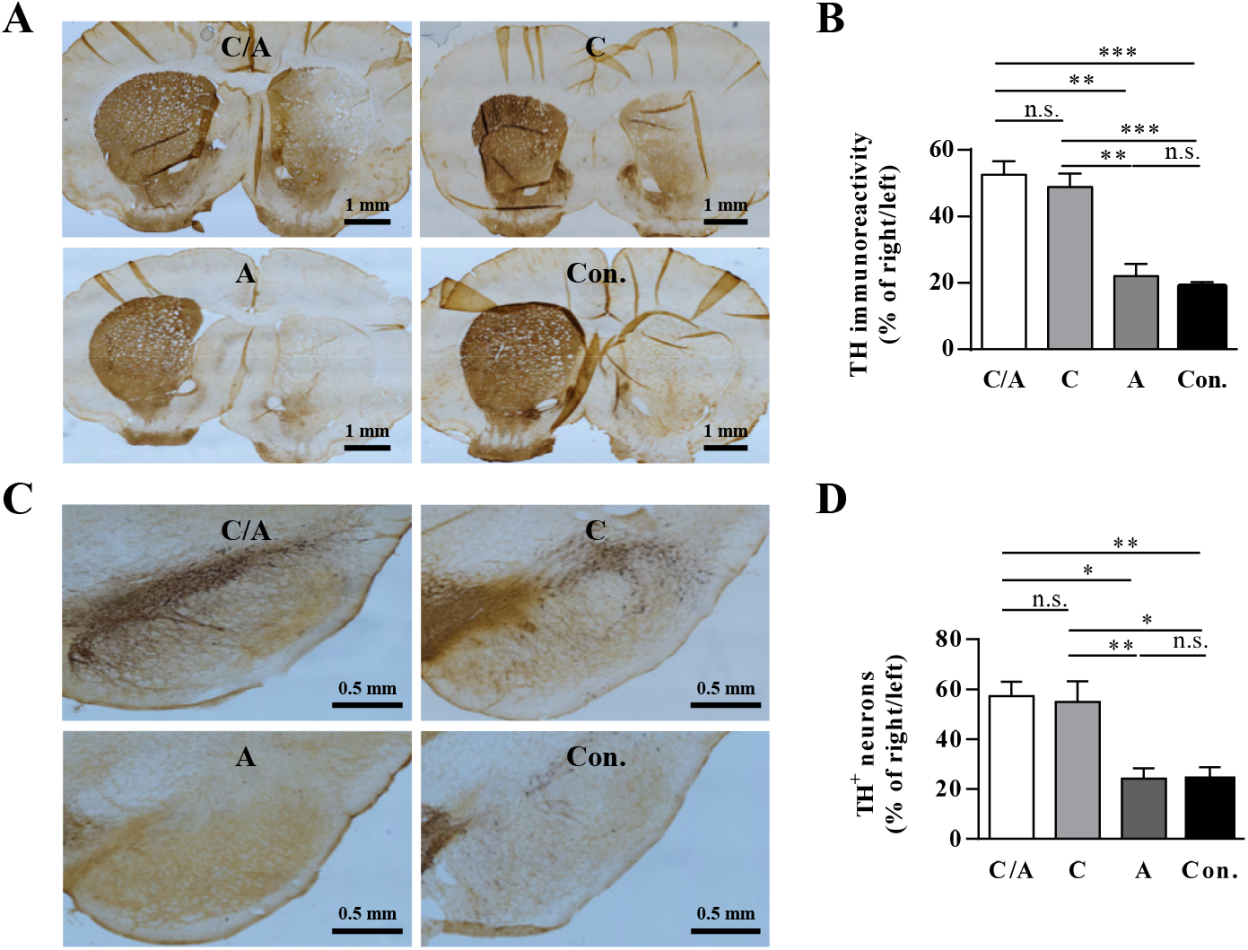
TH levels in the striatum and SNpc of PD rats at the late stage of the disease course after treatment. (A) Representative images of TH immunostaining of the striatum from 4 rats/group. (B) Quantification of TH immunoreactivity in the striatum. (C) Representative images of TH immunostaining of the SNpc from 4 rats/group. (D) Quantification of TH^+^ neurons in SNpc. ‘C/A’, ‘C’, ‘A’ and, ‘Con.’ represent ‘CDNF/AADC group’, ‘CDNF group’, ‘AADC group’ and ‘control group’, respectively, in (B) and (D); and the significant differences were calculated by one-way ANOVA. *P < 0.05, **P < 0.005, ***P < 0.001.

Immunostaining of the rat striatum and SNpc for TH and striatal dopamine quantification were conducted to examine the effects of the combination of AAV8-CDNF and AAV8-AADC. Rats administered AAV8-CDNF alone or the combination of AAV8-CDNF and AAV8-AADC showed stronger TH^+^ staining in the striatum (Fig 6A and 6B) of the lesioned side, while rats treated only with AAV8-AADC had no increase of TH^+^ fibers in the striatum of the lesioned side compared with AAV8-RFP group rats. Similarly, TH^+^ neurons of the SNpc (Fig 6C and 6D) in the lesioned side remained greater in rats administered AAV8-CDNF or the combination vectors, while TH^+^ neurons were rarely seen in the AAV8-AADC group and vehicle group.

**Fig 7:**
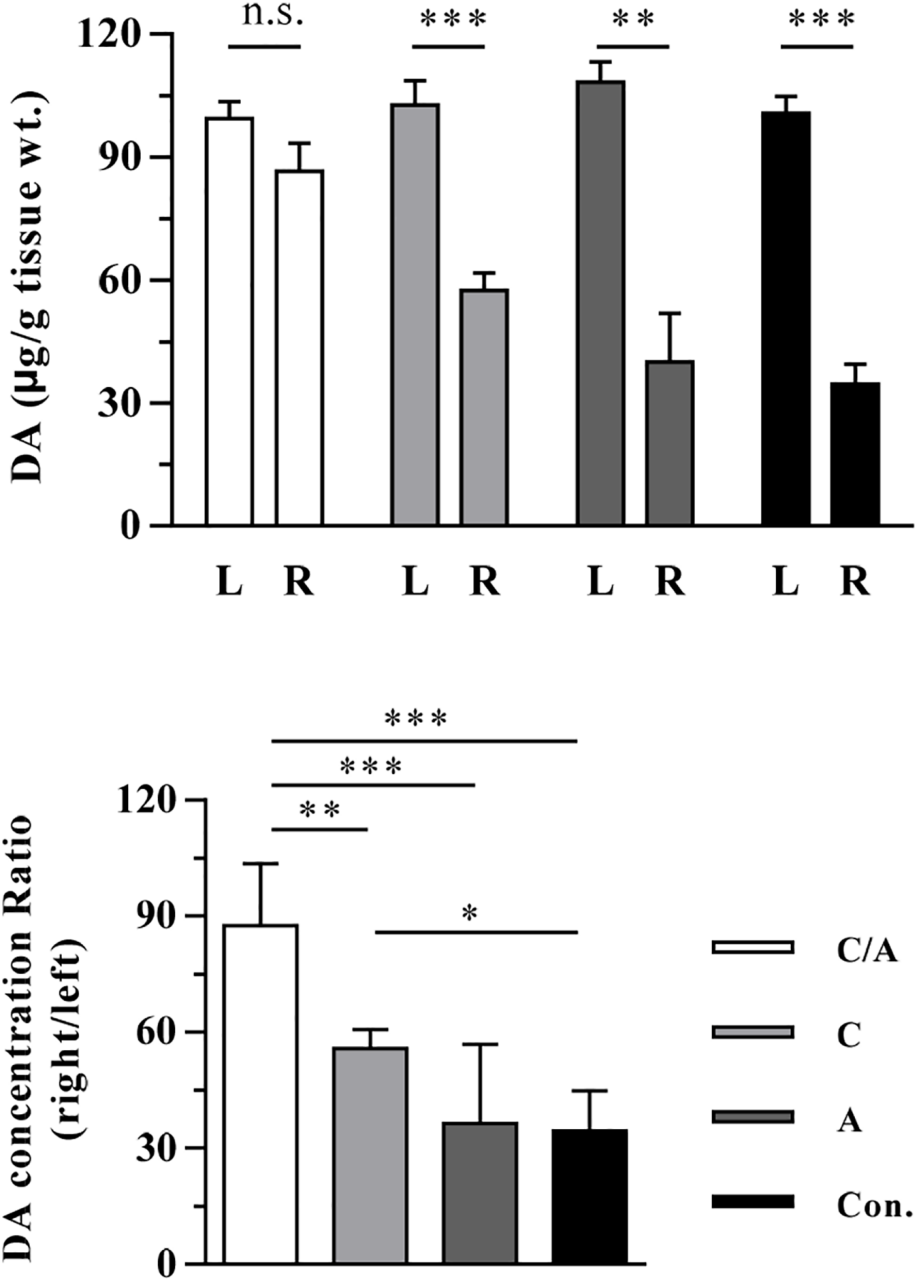
Striatal DA detection of PD rats at the late-stage of the disease course after treatment. The striatal DA was detected via HPLC. Four to five rats per group were analyzed. The DA concentration in the left (L) striatum and the right (R) are shown as micrograms per gram of tissue. Sigificant differences were analyzed by upaired t-test for the comparison between L and R. The ratios of DA in the right striatum to the left were calculated, and the results were analyzed by one-way ANOVA. *P < 0.05, **P < 0.005, ***P < 0.001. ‘C/A’, ‘C’, ‘A’ and, ‘Con.’ represent ‘CDNF/AADC group’, ‘CDNF group’, ‘AADC group’ and ‘control group’, respectively.

The dopamine concentration of the lesioned side in the brain directly determines the motor disorder of PD rats. Thus, we detected dopamine levels in the rat striatum of diverse groups by HPLC. As shown in Fig 7, the dopamine level in the striatum of the lesioned side returned to an average level of 87.5%, equivalent to that in the normal side in the combination therapy group. Meanwhile, the average dopamine level was increased to 57.0% (lesioned side/normal side) in the AAV-CDNF group, and it was approximately 35.0% (lesioned side/normal side) in the control group and AAV8-AADC group.

In summary, regardless of the fact that a single AAV8-AADC administration was unable to increase the dopamine level in the brain of severely lesioned PD rats due to low levodopa production from the few functional dopaminergic neurons, it could enhance the dopamine synthesis when combined with AAV8-CDNF administration.

## Discussion

CDNF has been proven to be an efficient treatment in the 6-OHDA-induced PD rat model and is considered to be a potential alternative neurotrophic factor for PD [21–23, 27, 28]. However, it may be not suitable for future application to clinical therapy when administered in the form of purified protein. Considering the generally limited half-life of proteins *in vivo*, the protection afforded by the CDNF protein may only be temporary and certainly would not last for several years. CDNF protein typically has been infused into the animal brain to study its neuroprotective effect [22, 26]. Using AAV to mediate long-term *CDNF* gene expression to provide lasting protection for dopaminergic neurons would be beneficial for PD patients. The rAAV8 serotype has been demonstrated to be relatively more efficient compared with rAAV2, rAAV5, rAAV9 and some other serotypes [29]. Thus, the rAAV8 vector may have the potential to generate a therapeutic level of the target protein when used for transgene expression. The rAAV8 vectors constructed in this study modulated transgene expression efficiently (Fig 1A). Viral vector-mediated GDNF overexpression has been reported to downregulate the striatal TH and cause aberrant sprouting in the downstream target nuclei in rodent models [30–32]. This finding gave us caution in evaluating AAV8-CDNF. Similar to the results of Ren *et*. *al*. [27], we found that our AAV8-CDNF-mediated *CDNF* gene transfer did not influence the TH expression in normal rats (Fig 1C).

As the 6-OHDA-induced PD rat model used in initial studies [22] generally showed modest lesions (with less than 50.0% TH loss), further studies are needed to investigate the therapeutic efficacy of CDNF in late PD. Few publications have directly compared the effect of CDNF in rat models with varying degrees of neuronal loss. The current study aimed to evaluate the effect of CDNF on PD rats with mild and severe lesions by using the classical 6-OHDA-induced PD rat model [33, 34]. Even though the 6-OHDA-induced rat model has the limitation of being unable to produce Lewy bodies, the rats with different lesions somewhat mimic the condition of PD patients with distinct neuronal loss at different stages.

Óscar *et al*. found that striatal CDNF overexpression by lentiviral vectors did not improve drug-induced rotational behavior or protect the dopaminergic system significantly in a severely lesioned rat model [28]. By contrast, Ren *et al*. reported that AAV2-mediated expression of CDNF in the striatum efficiently protected the TH^+^ cells and surprisingly improved the drug-induced rotational behavior of PD rats and raised the level of TH^+^ neurons in the SNpc to 77.0% of the control side, when AAV2-CDNF was administered 6 weeks post lesion with 6-OHDA [27]. Our results showed that the AAV8-CDNF-mediated striatal CDNF expression significantly increased TH levels in the striatum (85.5% of the control side) and SNpc (87.7% of the control side) when given at 2 weeks after inducing the lesion, but it had a limited effect (approximately 63.1% TH+ neurons in SNpc of the lesioned side compared to the control side) when given to severely lesioned rats (5 weeks post lesion). Interestingly, different outcomes were obtained from three studies using three types of viral vectors. Although rAAV8, rAAV2 and lentivirus are efficient vectors for gene delivery, comparing their efficacies across studies is difficult. Different 6-OHDA injection sites and treatment starting times in the studies may also account for differences in results.

In this study, AAV8-CDNF administration had a limited therapeutic effect in severely lesioned PD rats, as reflected by the failure to sufficiently restore the dopaminergic neurons and motor function (Figs 3 and 4). Possible explanations for this result may involve difficulties in restoring severely damaged dopaminergic neurons and few terminals being able to efficiently transport CDNF to the neuronal cell bodies [35–37]. Thus, CDNF may have a moderate effect in late-stage PD patients if applied in clinical trials. Based on this consideration, a strategy of combining CDNF with neurotrophic effects and an enzyme related to dopamine synthesis was explored in severely lesioned PD rats to better improve the behavioral symptoms. AADC has been shown to efficiently improve motor functions of PD patients in clinical trials [5, 38]. However, restoration of AADC activity alone still requires continued levodopa treatment to increase dopamine release in late PD due to low production of levodopa from the few dopaminergic neurons [4]. In the current study, severely lesioned rats administered AAV8-CDNF showed partial neuronal recovery; that is, levodopa could be produced by the surviving dopaminergic neurons. Thus, the combination with AADC to further enhance the dopamine synthesis may optimize the motor improvement provided by CDNF. A combination strategy with AAV8-CDNF and AAV8-AADC was developed, with the expectation of gaining a better improvement in motor function. Based on observations of motor improvement (Fig 5), TH recovery (Fig 6) and dopamine increase (Fig 7) in rats of the combination group, AADC demonstrated the ability to enhance dopamine synthesis with the joint use of CDNF, while it alone did not increase the dopamine levels or improve the motor disorders of severely lesioned rats. The combination of CDNF and AADC is a new potential alternative strategy to replace the therapy of AADC overexpression with continuous levodopa administrations.

In summary, we constructed 6-OHDA-induced PD rats with mild lesions and severe lesions as models of early PD and late PD, respectively. The therapeutic efficacy of CDNF was evaluated in the PD rats at different stages, and a combination strategy was tested in the severely lesioned rats. The results may help to further understand the therapeutic effects of CDNF and even offer a basis for future clinical application of AAV8-CDNF. Furthermore, a new combination strategy of CDNF and AADC was presented as a potential hopeful therapeutic strategy for late-stage PD patients. However, this study along with other studies using the 6-OHDA rat model can only initially explore the therapeutic effects of CDNF, since this model does not produce Lewy bodies and therefore cannot perfectly mimic clinical PD in humans. Thus, more direct evidence in PD animal models with Lewy bodies will need to be found before applying CDNF in clinical trials.

## Supporting information

S1 Fig Experimental design. (A) The schematic diagram of the injection sites of 6-OHDA and rAAV8 vectors. (D) The schematic diagram of the whole experimental design. Rats with rotation behavior were selected out at 10 days post 6-OHDA lesion for the following treatment. Early-treatment (n = 16) was carried out at 2 weeks post lesion, when the PD rats were with mild symptoms. Late-treatment (n = 14) was operated at 5 weeks post lesion, when PD rats had severe symptoms. The control group with AAV8-RFP administration contains 14 rats. Rotation behavior was tested every two weeks post treatment. At the end of the experiment, rats were killed for immunochemical staining or DA detection.
(TIF)

S2 Fig AAV8-AADC mediated AADC expression in the rat striatum. AAV8-AADC mediated AADC expression in the striatum was detected by immuno-histochemical staining at 2 weeks post viral injection.
(TIF)

S3 Fig Rotational behavior after 6-OHDA lesion. Rats (n = 5) were tested the rotational behavior induced by apomorphine (2mg/kg) at every week post 6-OHDA lesion.
(TIF)
