# Supplementary figures and images for: Therapeutic efficacy of AAV8-mediated intrastriatal delivery of human cerebral dopamine neurotrophic factor in 6-OHDA-induced parkinsonian rat models with different disease progression
Source: PLoS One. 2017 Jun 16;12(6):e0179476. doi: 10.1371/journal.pone.0179476 (PMC5473573; doi:10.1371/journal.pone.0179476)

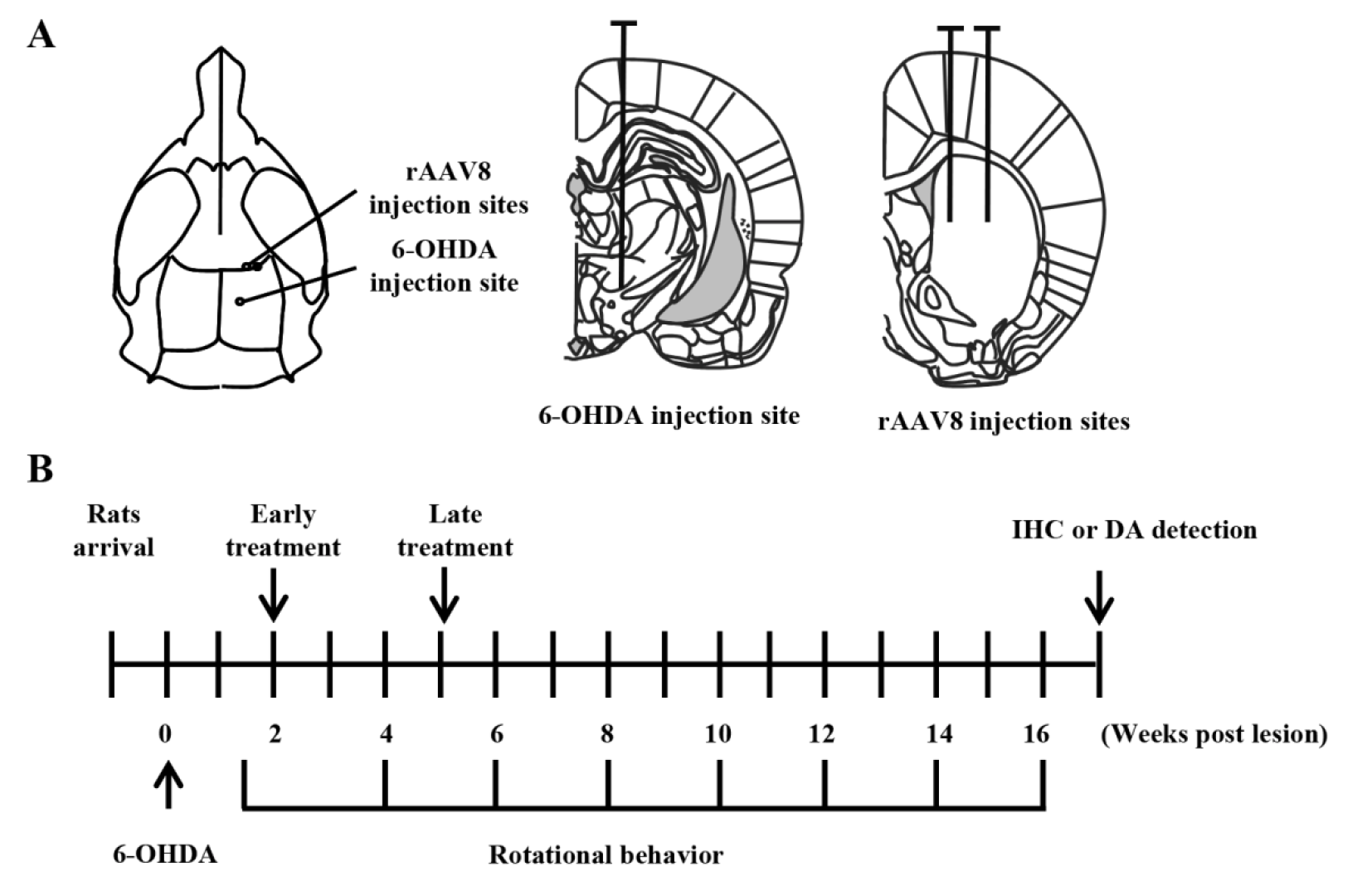

Supplement: S1 Fig — (A) The schematic diagram of the injection sites of 6-OHDA and rAAV8 vectors. (D) The schematic diagram of the whole experimental design. Rats with rotation behavior were selected out at 10 days post 6-OHDA lesion for the following treatment. Early-treatment (n = 16) was carried out at 2 weeks post lesion, when the PD rats were with mild symptoms. Late-treatment (n = 14) was operated at 5 weeks post lesion, when PD rats had severe symptoms. The control group with AAV8-RFP administration contains 14 rats. Rotation behavior was tested every two weeks post treatment. At the end of the experiment, rats were killed for immunochemical staining or DA detection. (TIF) [file pone.0179476.s001.tif]

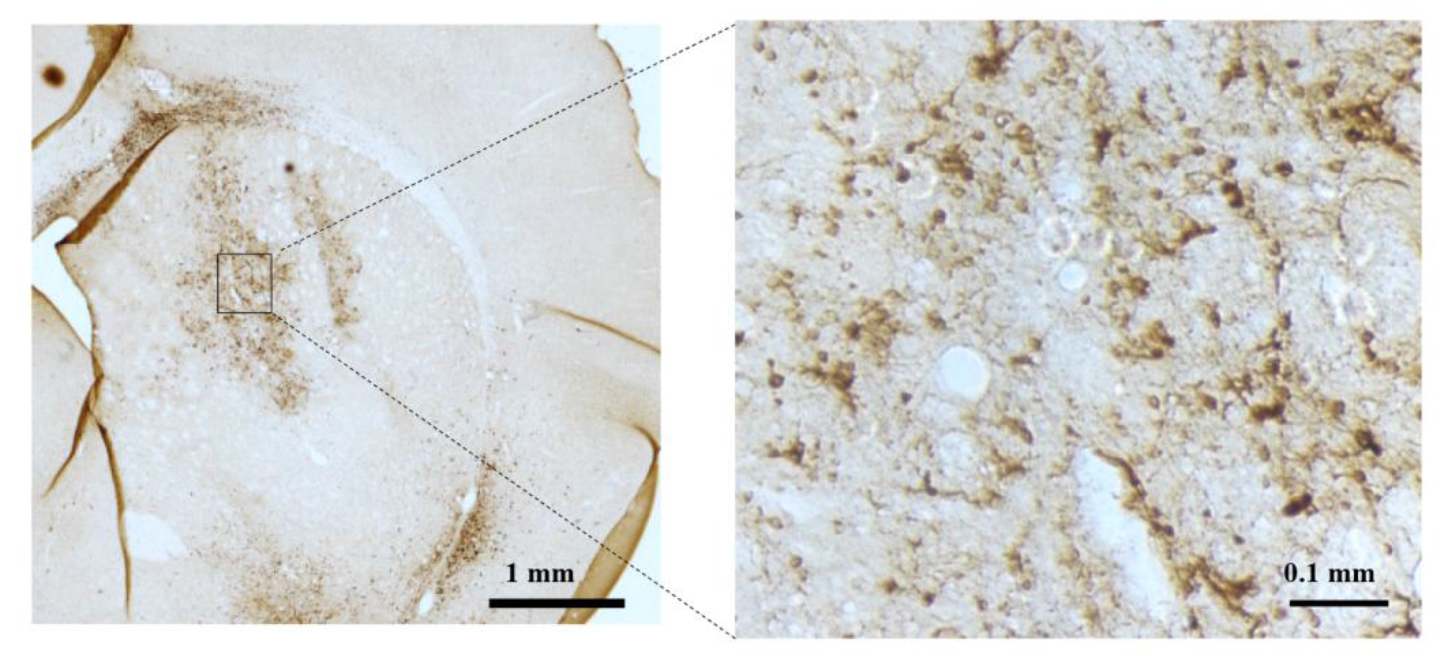

Supplement: S2 Fig — AAV8-AADC mediated AADC expression in the striatum was detected by immuno-histochemical staining at 2 weeks post viral injection. (TIF) [file pone.0179476.s002.tif]

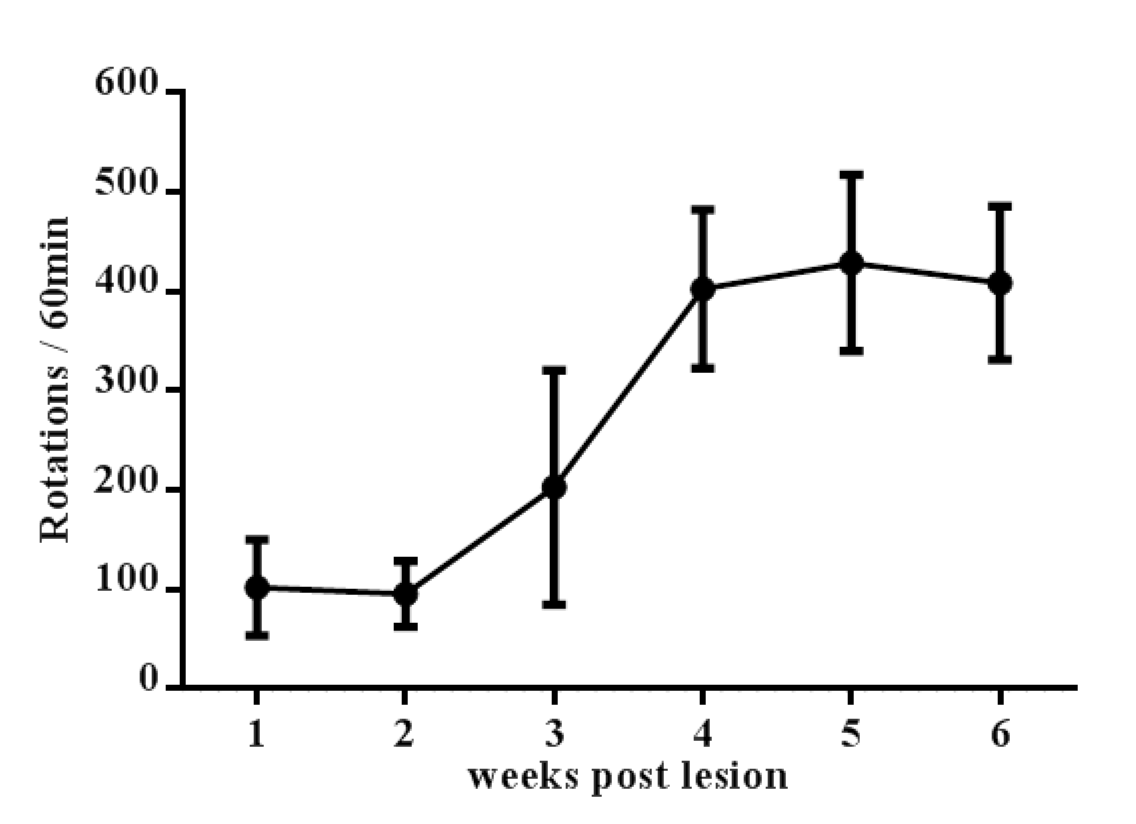

Supplement: S3 Fig — Rats (n = 5) were tested the rotational behavior induced by apomorphine (2mg/kg) at every week post 6-OHDA lesion. (TIF) [file pone.0179476.s003.tif]
